# The impact of endolymphatic hydrops on wideband acoustic immittance and otoacoustic emissions in guinea pigs

**DOI:** 10.3389/fneur.2025.1444928

**Published:** 2025-01-23

**Authors:** Huan Lin, Xuanyi Li, Huiying Zhang, Yi Mu, Xi Wang, Naveena Konduru, Renlong Ji, Wen Liu, Zhao Fei, Wen Jiang, Yuehua Qiao

**Affiliations:** ^1^Jiangsu Auditory Engineering Research Center, The Second College of Clinical Medicine, Xuzhou Medical University, Xuzhou, China; ^2^ENT Department, The Affiliated Hospital of Xuzhou Medical University, Xuzhou, China; ^3^The First College of Clinical Medicine, Xuzhou Medical University, Xuzhou, China; ^4^Centre for SLT and Hearing Sciences, Cardiff School of Sport and Health Sciences, Cardiff Metropolitan University, Cardiff, United Kingdom

**Keywords:** wideband acoustic immittance, endolymphatic hydrops, ABR, DPOAE, guinea pigs

## Abstract

**Background:**

Inner ear disorders, such as EH, commonly lead to hearing loss and vestibular dysfunction. EH is particularly prevalent in various inner ear diseases, including Meniere’s disease. We aimed to evaluate the effects of EH on WAI and OAEs.

**Objective:**

This study explores the potential of wideband acoustic immittance (WAI) and otoacoustic emissions (OAEs) for the early detection of changes in acoustic transmission associated with vasopressin-induced endolymphatic hydrops (EH) in a guinea pig model.

**Methods:**

We induced EH in guinea pigs via daily intraperitoneal injections of arginine vasopressin over 14 consecutive days. Auditory function was assessed using Auditory Brainstem Responses (ABR), while changes in sound energy transmission were measured using WAI and Distortion Products Otoacoustic Emissions (DPOAE).

**Results:**

Increased ABR thresholds in EH models were statistically significant (*p* < 0.05). After 14 days of EH induction, absorbance at 1 kHz significantly increased, whereas it significantly decreased at 4 kHz and 6 kHz (*p* < 0.05). DPOAE measures, both magnitude and phase, showed no significant changes (*p* > 0.05).

**Conclusion:**

WAI demonstrates greater sensitivity than DPOAE in the early detection of acoustic transmission alterations in EH models, suggesting its utility as a diagnostic tool in early-stage inner ear disorders.

## Introduction

Endolymphatic hydrops (EH) is characterized by the excessive accumulation of endolymph within the inner ear and is widely regarded as a hallmark pathology of Meniere’s disease (1). EH is also associated with other auditory disorders, such as large vestibular aqueduct syndrome (2). Although the exact relationship between EH and its clinical manifestations remains partially understood, it is hypothesized that EH disrupts auditory and vestibular functions primarily through increased endolymphatic pressure. This condition occurs when the distension of the membranous labyrinth increases endolymphatic system pressure, creating a pressure differential between the perilymphatic and endolymphatic compartments (3). Such pressure imbalances may induce mechanical stress on labyrinthine structures, impairing both auditory and vestibular function (3).

Despite a variety of diagnostic tests available for EH, a definitive gold standard remains elusive. Established methods, such as vestibular evoked myogenic potentials (4), electrocochleography (5), and otoacoustic emissions (OAEs) (6), provide valuable insights but are limited in their sensitivity and specificity. Gadolinium-enhanced MRI enables direct visualization of EH and is increasingly recognized as a diagnostic tool for Meniere’s disease, supported by its inclusion in clinical guidelines such as those proposed by the Japan Society for Equilibrium Research (7). However, gadolinium-based MRI requires intravenous contrast administration, rendering it minimally invasive. Moreover, its widespread clinical application is constrained by high costs, limited accessibility, and its inability to assess dynamic biomechanical processes, such as pressure-induced changes in the inner ear. These limitations underscore the need for non-invasive, cost-effective diagnostic approaches that can provide accurate insights into the biomechanical and physiological status of the inner ear.

Wideband acoustic immittance (WAI) is an emerging diagnostic tool that measures the mobility of the middle ear across a broad frequency range, providing valuable frequency-specific insights into acoustic transmission (8). Clinically, WAI has been extensively applied to assess middle-ear conditions such as otitis media with effusion (9), otosclerosis (10, 11), and ossicular chain disruptions (12), often demonstrating higher diagnostic sensitivity compared to traditional tympanometry. Animal studies have validated WAI’s effectiveness, with Guan et al. demonstrating its ability to detect sound energy absorbance changes in chinchilla models of acute otitis media, highlighting its fluid sensitivity (13). Margolis et al. showed interspecies consistency between chinchillas and humans, supporting its translational relevance (14), while Akinpelu et al. confirmed its precision in detecting middle-ear impedance changes (15). Furthermore, WAI has proven useful in diagnosing inner ear pathologies linked to ‘third window’ phenomena, such as superior semicircular canal dehiscence (16), Meniere’s disease (17) and large vestibular aqueduct syndrome (18–20). In these conditions, WAI detects distinct frequency-specific changes caused by pressure-induced alterations in inner ear mechanics, making it a promising tool for evaluating disorders associated with abnormal pressure dynamics (18).

Guinea pigs are widely recognized as an established model for auditory research due to the similarity of their cochlear anatomy and mechanics to humans (21–25). Previous studies using guinea pigs have successfully replicated EH-like conditions, providing insights into the pathological mechanisms of EH and its effects on auditory structures (23, 24). Specifically, these studies demonstrated that EH models in guinea pigs induce frequency-specific changes in auditory brainstem response (ABR) thresholds and acoustic transmission properties, offering a controlled platform to investigate inner ear pathologies (23, 24).

While clinical observations suggest alterations in acoustic properties in patients with Meniere’s disease, the role of WAI in detecting and monitoring EH has not yet been fully investigated (17). This study leverages guinea pig models to assess the diagnostic potential of WAI in detecting EH-induced changes in acoustic mechanics, particularly in relation to frequency-specific alterations in middle-and inner-ear mechanics.

Our aim was to assess the sensitivity of WAI and OAEs in detecting EH-induced biomechanical changes in the middle and inner ear. While this study focuses on animal models, the findings contribute to our understanding of EH’s acoustic manifestations and provide insights into the potential utility of WAI as a non-invasive diagnostic tool. This research enhances our understanding of EH pathophysiology and establishes a foundation for applying WAI in EH diagnosis and treatment monitoring, highlighting the need for translational studies to validate these findings in clinical settings.

## Materials and methods

### Experimental design

**Figure 1:**
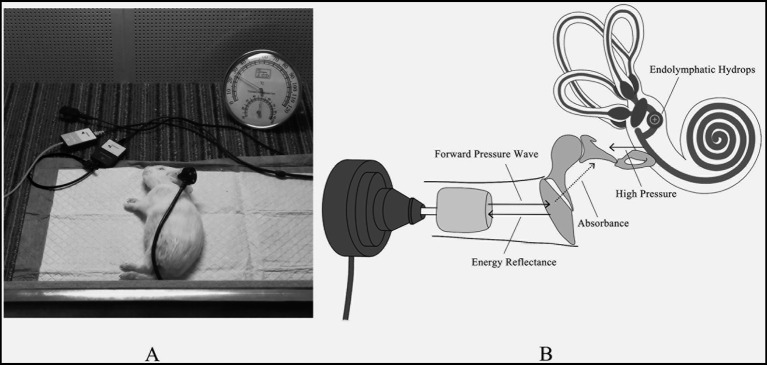
**(A)** All measurements were tested in a constant temperature soundproof booth. **(B)** The schematic diagram of WAI detecting EH.

The experimental protocol received approval from the Experimental Animal Ethics Committee of Xuzhou Medical University, Jiangsu Province, China (Ethics No: L20210226392). Animals exhibiting hearing thresholds above 35 dB SPL, as determined by auditory brainstem responses (ABR), were excluded to avoid preexisting auditory abnormalities. Twelve healthy albino guinea pigs, weighing between 200 and 350 grams, participated in the study. Seven guinea pigs in the experimental group received an intraperitoneal injection of 10 μg/kg desmopressin acetate (DL123296, CAS: 16679–58-6, Aladdin, China) daily for 14 consecutive days to induce endolymphatic hydrops (EH). The control group received physiological saline. The dosage and duration were selected based on established protocols from prior studies that successfully induced EH in guinea pigs using vasopressin or desmopressin, providing a reliable and reproducible model for inner ear research (23–27). This protocol ensures sufficient stimulation of the arginine vasopressin (AVP)-aquaporin pathway to replicate pathological features of EH while minimizing adverse effects on the animals. Detailed justification for the dosage and protocol is provided in Supplementary Table 1. Measurements of auditory brainstem response (ABR), distortion product otoacoustic emissions (DPOAE), and wideband acoustic immittance (WAI) were performed prior to the experiment and subsequently at 7 and 14 days within a soundproof booth (Figure 1). Guinea pigs were anesthetized with sodium pentobarbital (40 mg/kg) and received additional anesthesia as needed to maintain areflexia throughout the experiment.

**Figure 2:**
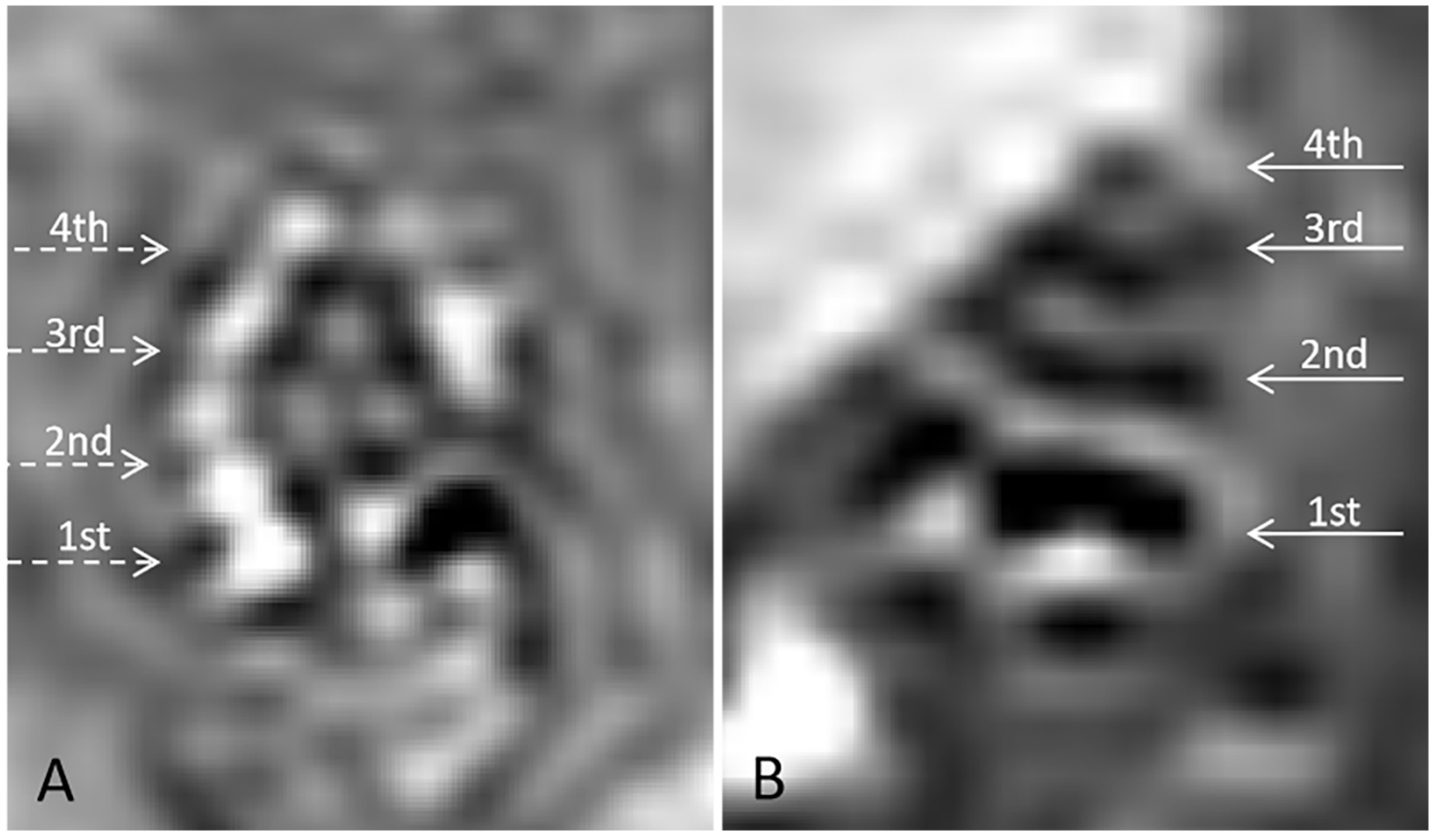
Gadolinium-enhanced 3D Real-IR MRI imaging of cochlear structures in guinea pigs. **(A)** Control group: 3D Real-IR imaging of a normal guinea pig cochlea showing distinguishable signals in the perilymphatic and endolymphatic spaces, with a normal proportion maintained between these compartments. **(B)** Experimental group (DDAVP-induced EH): 3D Real-IR imaging reveals significant enlargement of the endolymphatic space, accompanied by a relative reduction in the perilymphatic space, indicative of endolymphatic hydrops (EH). The cochlea is segmented into distinct regions: 1st represents the basal turn, 2nd indicates the second turn, 3rd refers to the third turn, and 4th represents the apical return.

To confirm the presence of EH, gadolinium-enhanced magnetic resonance imaging (MRI) was conducted on the experimental group 24 h after the 14-day desmopressin induction period. Bilateral tympanic injections of gadolinium contrast agent (gadopentetate dimeglumine) were administered prior to MRI scanning. The images were compared with those from control animals, revealing significant endolymphatic distension in the experimental group. These results validate the successful induction of EH and provide visual evidence of the pathological changes (Figure 2).

### ABR measurements

ABR thresholds were recorded using the Neuro-Audio NET system version 1.0.103.3 (Neurosoft, Ivanovo, Russia). Needle electrodes were positioned subcutaneously at the vertex (positive), bilateral mastoids (negative), and the nose (ground). Click stimuli with alternating polarity and a duration of 0.1 ms were presented, starting at 90 dB SPL and decreasing in 5 dB increments. Thresholds were primarily identified by the presence of waves I, III, and V of the ABR.

### Distortion products otoacoustic emissions measurements

DPOAEs were assessed using Mimosa Acoustics hardware and software (DPOAE module C 1.1.0.2, HearID V5.1.9.3). Measurements were conducted across 10 logarithmically spaced frequencies for f_2_ ranging from 516 to 9,984 Hz, maintaining a fixed f_2_/f_1_ ratio of 1.22, with intensities set at 65 dB SPL for L_1_ and 55 dB SPL for L_2_. DPOAE responses were considered normal if the signal-to-noise ratio (SNR) exceeded 6 dB above the ambient noise level.

### WAI measurements

Reflectance and immittance parameters were assessed using an Etymotic ER-10C probe coupled with Mimosa Acoustics software (MEPA3 module 3.5.2.4, HearID V5.1.9.3). Before each testing session, the WAI equipment was calibrated using a four-cavity calibration device. The probe was fitted with the smallest available rubber ear tips (size 03) and placed in the external ear canal. WAI measurements utilized a chirp stimulus at 60 dB SPL, spanning 248 points from 0.2 Hz to 6 kHz. Statistical analyses focused on octave bands from 250 to 4,000 Hz, with the maximum frequency analyzed being 6,000 Hz. The resonance frequency (RF) was determined directly from the impedance magnitude (|*Z(f)*|) data obtained during WAI testing. RF was defined as the frequency at which (|*Z(f)*|) exhibited a sharp dip, reflecting the Helmholtz resonance effect (28). This corresponds to the point where stiffness-dominated and mass-dominated reactances cancel each other, resulting in minimal impedance magnitude.

### Statistical analyses

Statistical analyses were conducted using SPSS version 22.0 (IBM, Armonk, NY, United States). Data are presented as means ± standard errors unless specified otherwise. The generalized estimating equation (GEE) was employed to analyze repeated measurements and did not presume a normal distribution of data. This model was used to compare ABR thresholds, absorbance, and impedance magnitudes across the three stages in the EH groups. An independent samples *t*-test and nonparametric tests were applied to compare absorbance between EH ears before injection and control ears after 14 days. A *p*-value of ≤0.05 was considered statistically significant.

## Results

### Auditory thresholds

**Figure 3:**
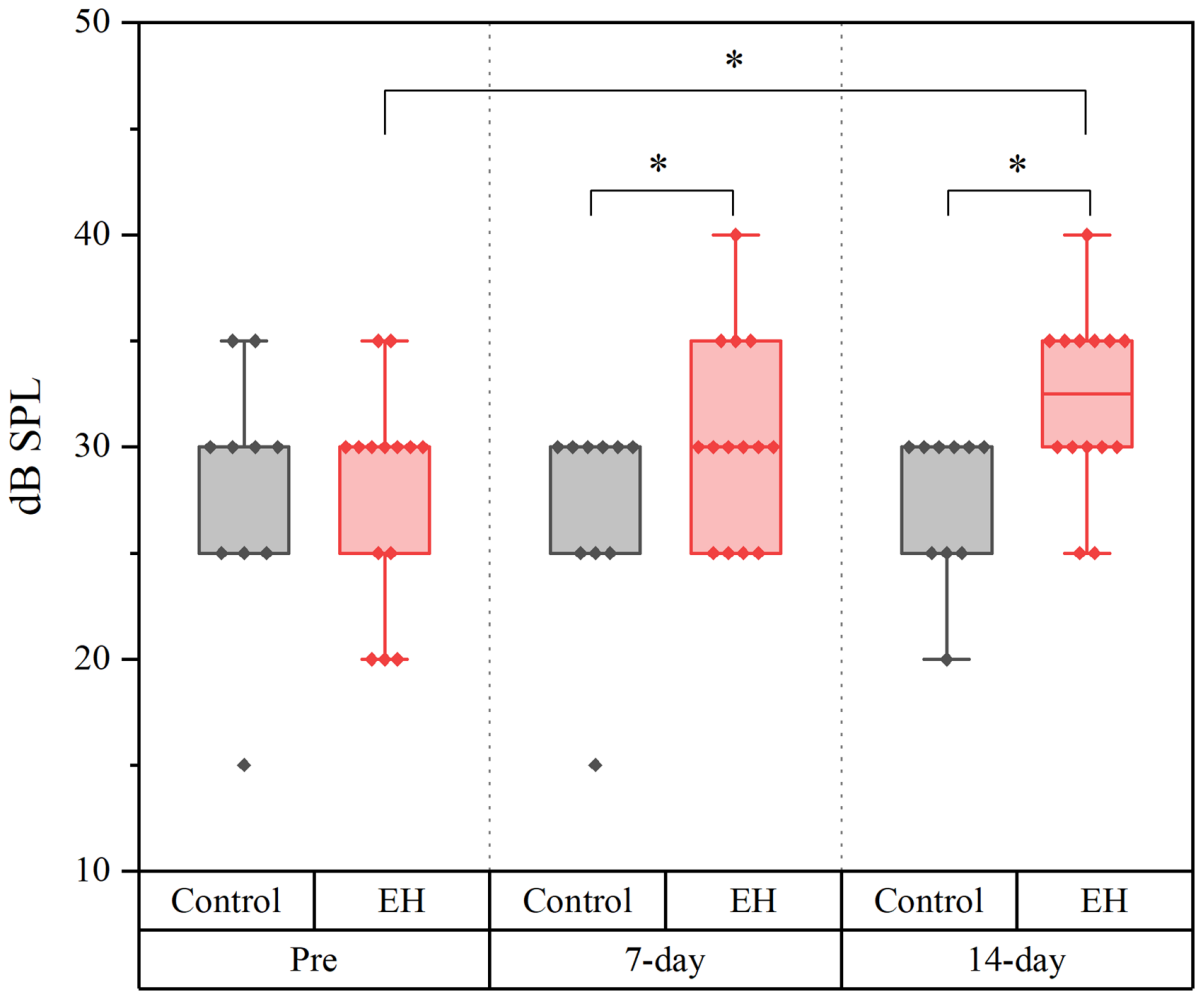
Box plot illustrating the median, the 25% upper and lower quartiles, and the minimum and maximum of the air conduction thresholds of the eight ears, before experiment (pre), 7-and 14-day EH groups.

The ABR thresholds were consistently higher in the EH group compared to controls, average increasing by 5 dB after 7 days (*p* < 0.05) and 10 dB after 14 days (*p* < 0.05), as shown in Figure 3. Statistical analysis using a Generalized Estimating Equation (GEE) confirmed significant differences in ABR thresholds between the groups due to EH. Pairwise comparisons with Bonferroni adjustment revealed that the 14-day EH group exhibited a significantly elevated ABR threshold (32.14 ± 1.14 dB) compared to the pre-EH condition (27.86 ± 1.36 dB; *p* < 0.05).

### Distortion products otoacoustic emissions

**Figure 4:**
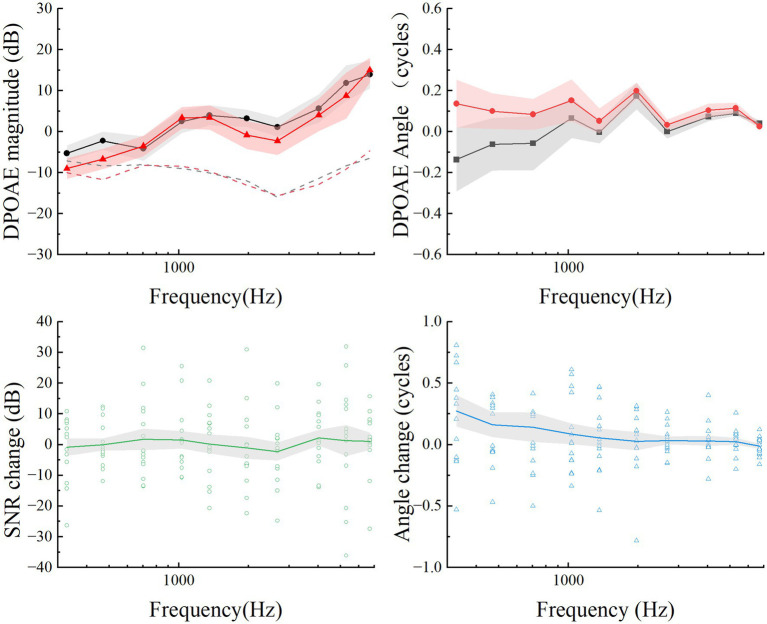
Summary of mean DPOAEs (upper plots) and differences (lower plots) for the pre-EH and EH 14-day groups. The mean DPOAE magnitude and angles for pre-EH and EH 14-day ears are calculated as function of the frequency *f*_2_ and plotted in solid points and lines. The mean maximum noise floor at each frequency is plotted in dashed lines on the magnitude plot. For each subject, open points are the mean differences at each frequency. The gray shaded regions represented Error bars are ±1 standard deviation.

DPOAEs measurements were conducted to assess cochlear function, especially out hair cell integrity, in the 14-day EH group. As illustrated in Figure 4, variations in DPOAEs magnitude and phase were minimal, with no significant differences observed between stages (*p* > 0.5). A comparison of SNR differences between the pre-EH and 14-day EH groups revealed negligible changes, consistently approaching zero as shown in the lower plots of Figure 4.

### Wideband absorbance and resonance frequency

**Figure 5:**
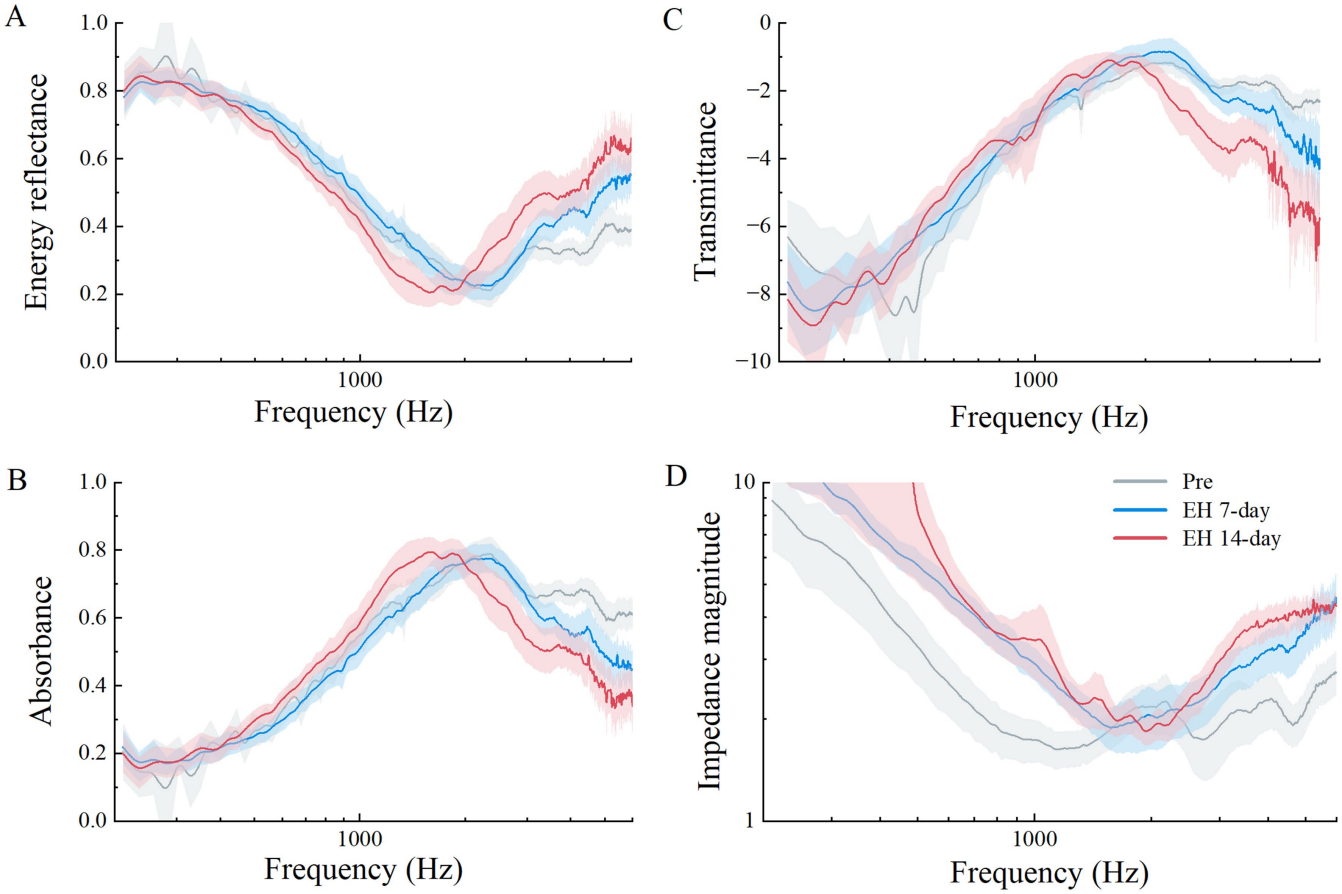
Acoustic properties measured between three experiment stages in the EH group, means in solid lines (gray for pre-EH ears, red for EH 7-day ears, and blue for EH14-day ears), shaded regions represent the mean ± 1 standard error. **(A)** Power reflectance (|*R*|^2^) in percent, **(B)** power absorption (1 – |*R*|^2^) in percent, **(C)** transmittance (10 × log_10_[1 – (|*R*|^2^)]) in decibels, and **(D)** impedance magnitude (|*Z*|).

Figure 5 illustrates a comparison of various acoustic parameters critical for assessing the middle ear (ME) status. These include power reflectance (|*R(f)*|^2^), power absorption (1 – |*R(f)*|^2^), transmittance (expressed in decibels as 10 × log [1 – |*R(f)*|^2^]), and impedance magnitude (|*Z(f)*|). The squared pressure reflectance magnitude, |*R(f)*|^2^, known as energy reflectance, indicates the proportion of sound energy reflected upon entering the ear canal. A value of 0 signifies complete absorption by the ear structures, whereas a value of 1 indicates total reflection. Resonance frequency (RF) was derived directly from the impedance magnitude (|*Z(f)*|) curves, representing the frequency at which (|*Z(f)*|) exhibited a sharp dip. This RF corresponds to the Helmholtz resonance effect, where stiffness-dominated and mass-dominated reactances cancel each other.

As shown in Figure 5A, the energy reflectance across three EH stages over frequencies from 0.2 to 6 kHz demonstrated that while the 7-day EH group mirrored the pre-EH group’s pattern, a noticeable increase occurred above 3 kHz. The 14-day EH group exhibited increased reflectance above 2 kHz but decreased levels between 1 and 2 kHz compared to earlier stages.

Figure 5B displays the absorbance data. Normal guinea pigs typically show a steady increase in absorbance at lower frequencies, peaking near 0.8 at 2.344 kHz, before sharply declining. In contrast, the EH groups exhibited lower absorbance rates above 3 kHz, with the 14-day EH group displaying higher absorbance between 1 and 2 kHz compared to normal ears. Statistical comparisons across the EH and 14-day control groups highlighted significant absorbance differences at 1, 4, and 6 kHz (*p* < 0.05), with no notable differences between pre-EH and control conditions (*p* > 0.05).

The transmittance data, presented in Figure 5C, translates absorbed power into a logarithmic scale. The transmittance curve for the 14-day group fell below that of both the pre and 7-day groups at frequencies above 2 kHz. Impedance magnitude (|*Z(f)*|), depicted in Figure 5D, Impedance magnitude (|Z(f)|), depicted in Figure 5D, showed significant deviations from normal across most frequencies in both the 7-day and 14-day EH groups. The RF values significantly increased in the EH groups compared to the pre-EH group (*p* < 0.05), with mean values of 1,239 Hz for the Pre-EH group, 1748 Hz for the EH 7-Day group, and 1785 Hz for the EH 14-Day group. However, no significant difference was observed between the EH 7-Day and EH 14-Day groups (*p* > 0.05), indicating that the increase in RF stabilized after 7 days of EH induction. Additionally, the 14-Day Control group exhibited no significant difference in RF compared to the pre-EH group (*p* > 0.05), confirming the preservation of normal middle ear mechanics in the absence of EH. These findings, highlighted in Figure 5D, suggest a progressive stiffening of the middle ear system due to EH, which stabilizes after 7 days (Table 1).

**Table 1:** List of *p* values from (1) GEE test on the WBA and RF at three stages in the EH group; (2) Independent-samples t-test on the WBA between pre-EH, EH 7-day, EH 14-day and control 14-day.

Frequency (kHz)	GEE *p-*value	Pre vs. EH 7-day	Pre vs. EH 14-day	EH 7-day vs. EH 14-day	Control 14-day vs. Pre
0.25	0.844	0.907	0.843	0.560	0.338
0.5	0.487	0.594	0.622	0.236	0.153
1	**0.001**	0.295	0.329	**0.001**	0.959
2	0.994	0.911	0.928	0.999	0.911
4	**0.030**	**0.042**	**0.010**	0.273	0.889
6	**0.025**	**0.049**	**0.007**	0.158	0.232
RF	0.054	**0.024**	**0.048**	0.878	0.283

Pre: normal ears before injection in EH group. GEE, Generalized Estimating Equation; WBA, Wideband Absorbance; EH, Endolymphatic Hydrops; RF, Resonance frequency.Bold values indicate statistically significant results (*p* < 0.05).

## Discussion

Our study has demonstrated significant changes in ABR thresholds in models of EH, marked by statistically significant increases. Following 14 days of EH induction, we noted a significant rise in absorbance at 1 kHz, alongside notable declines at 4 kHz and 6 kHz. These results highlight EH’s selective impact across different frequency ranges. In contrast, measurements of DPOAE, including both magnitude and phase, revealed no significant changes, which emphasizes DPOAE’s limited sensitivity in detecting early alterations in acoustic transmission due to EH. Notably, WAI exhibited greater sensitivity compared to DPOAE in identifying early acoustic changes, suggesting its potential as an effective diagnostic tool in the early detection of inner ear disorders.

**Figure 6:**
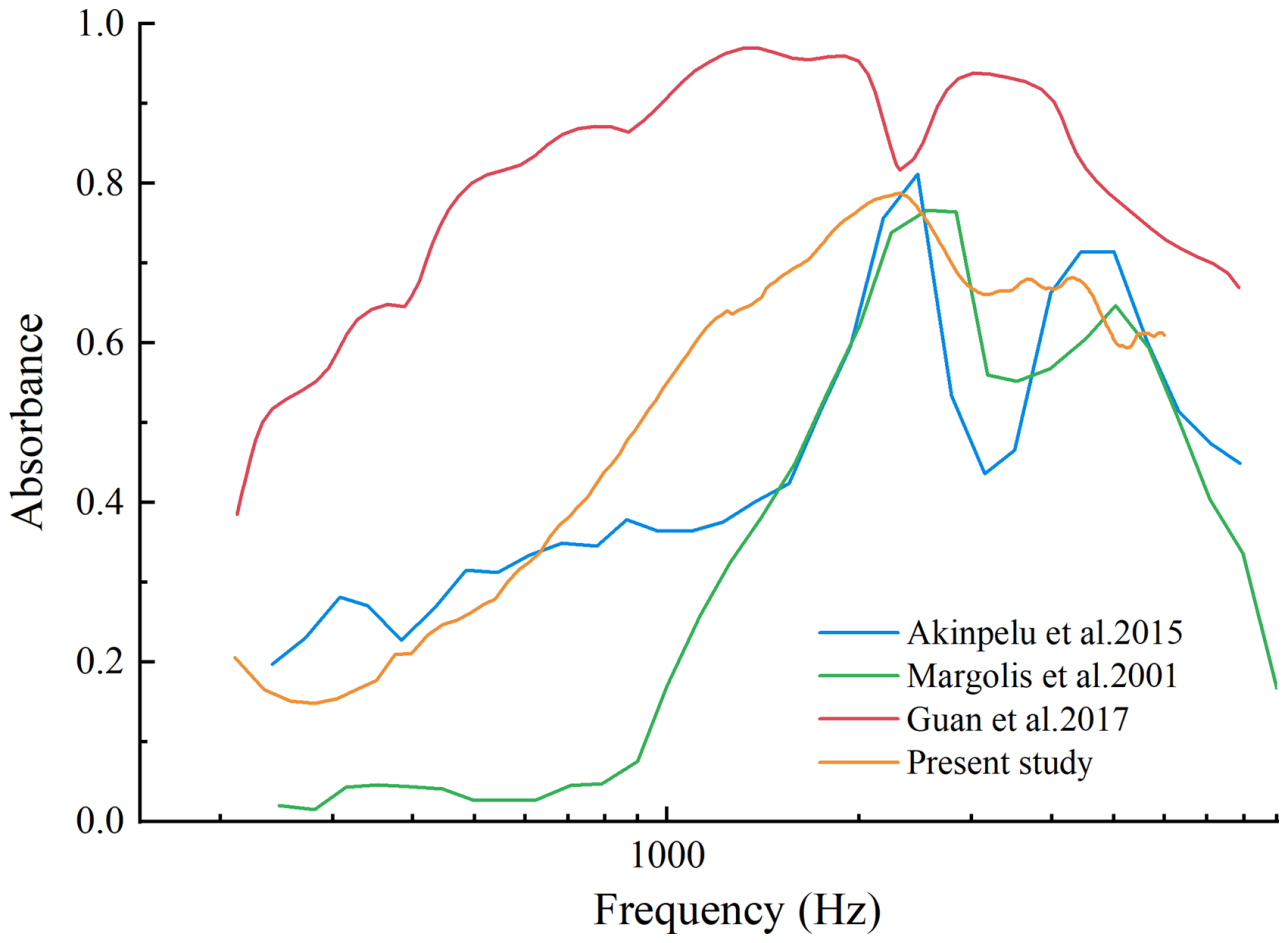
Absorbance curve from normal chinchillas’ ears in Margolis et al., 2001, Akinpelu et al., 2015, Guan et al., 2017, and normal guinea pigs’ ears of present study.

Guinea pigs, widely used in auditory research due to their physiological similarities to humans, provide valuable insights into EH-related changes in middle-ear mechanics. However, reports on WAI outcomes in guinea pigs remain limited. By transforming energy reflectance data into absorbance, our study allows for a direct comparison with previously documented chinchilla data (13–15), helping to bridge the gap between species-specific research findings (Figure 6). Despite using different measurement systems, the disparities between these systems are less significant than the variations observed between pathological and normal middle-ear conditions (29). Our findings show that guinea pigs and chinchillas display similar absorbance patterns, particularly low absorbance at lower frequencies and a characteristic V-shaped curve at higher frequencies. These findings enhance the translational relevance of guinea pig models while underscoring the importance of considering species-specific differences, such as variations in ossicular chain stiffness and resonance frequencies, when referring results to humans.

Our study also revealed significant changes in resonance frequency (RF), with RF markedly increasing in EH groups compared to the pre-EH group. The stabilization of RF changes by 7 days suggests a plateau in the stiffening of the middle ear system caused by EH. RF, defined as the frequency at which stiffness-reactance and mass-reactance are equal, serves as an indicator of changes in middle-ear mechanics (18). An increase in middle-ear stiffness elevates RF, while increased mass or reduced stiffness lowers it (18). In conditions like large vestibular aqueduct syndrome (LVAS), elevated RF reflects increased inner ear pressure, reduced stapes compliance, and heightened middle-ear stiffness (18). These mechanisms align with our findings in EH, where fluid retention likely altered tympano-ossicular dynamics, contributing to RF elevation.

These findings align closely with prior research on EH and its acoustic manifestations. Marshall et al. demonstrated vasopressin-induced EH using gadolinium-enhanced MRI and noted its impact on cochlear mechanics, findings consistent with the altered acoustic transmission observed in our study (23). Similarly, Jiang et al. reported fluid retention mediated by the AVP-aquaporin pathway, correlating with the reduced WAI absorbance at mid-to-high frequencies (24). The limited sensitivity of DPOAE observed in our study mirrors findings from other investigations. Valk et al. and Chihara et al. observed only minor alterations in DPOAE magnitude and phase in response to changes in inner-ear pressure in guinea pig models of EH (21, 22). Rotter et al. found that low-frequency DPOAE tests exhibited limited sensitivity compared to electrocochleography in diagnosing EH (30). These results emphasize the complementary rather than standalone role of DPOAE in EH diagnostics and highlight WAI’s superior capacity to detect frequency-specific acoustic changes, particularly in the context of chronic EH.

EH-induced alterations in middle-ear mechanics influence fundamental acoustic properties such as stiffness, mass, and friction. The observed increase in RF, along with changes in impedance magnitude, underscores the impact of EH on middle-ear stiffness and its potential as a diagnostic marker for inner ear disorders. Notably, these changes are consistent with clinical observations from human patients with EH, reinforcing the relevance of our animal model. The increased impedance magnitude in EH-affected guinea pigs suggests a novel parameter for detecting changes in inner ear pressure.

Our findings on WAI outcomes in guinea pigs—such as reduced absorbance at lower frequencies and the characteristic V-shaped curve—align closely with chinchilla data (13–15), highlighting inter-species consistency. The frequency-specific patterns observed in guinea pigs—reduced absorbance at mid-to-high frequencies—parallel those reported in clinical studies of Meniere’s disease (8), highlighting WAI’s potential for non-invasively diagnosing early-stage inner-ear disorders. The clinical utility of WAI lies not only in its diagnostic sensitivity but also in its practicality. WAI is non-invasive, requires minimal patient preparation, and provides rapid, frequency-specific data on middle-ear impedance, making it well-suited for outpatient and screening settings. By offering real-time functional insights into middle-ear mechanics, WAI complements traditional diagnostic methods and bridges the gap between anatomical imaging and physiological assessment. These attributes enhance its potential as a translational tool, leveraging guinea pig models to inform clinical diagnostics. However, inter-species anatomical and biomechanical differences, such as variations in ossicular chain stiffness and resonance frequencies, necessitate careful consideration when applying these findings to human contexts. Addressing these differences is essential for refining WAI protocols and ensuring consistent and accurate application in clinical practice.

Despite the strengths of this study, there are several limitations that warrant discussion. First, inter-individual variability in acoustic measurements, particularly in WAI and DPOAE, was not explicitly considered. Differences in cochlear anatomy, middle-ear mechanics, and the degree of EH development among subjects may have contributed to variability in the results. Variability in WAI absorbance profiles at specific frequencies may reflect differences in endolymphatic distension, as previously noted in EH studies (13–15). Second, the relatively small sample size (seven guinea pigs per group) aligns with previous research (22, 25) but limits the statistical power and generalizability of our findings. Additionally, while gadolinium-enhanced MRI confirmed EH induction in a subset of subjects, not all animals were verified for EH presence or severity. This lack of confirmation introduces potential variability in WAI and DPOAE measurements.

Future studies should incorporate larger sample sizes, post-induction confirmation techniques, and stratification of subjects based on EH severity to enhance the robustness of findings. Translational research validating WAI in human populations is essential to establish diagnostic thresholds and refine its clinical application for specific inner-ear disorders. Combining WAI with other non-invasive diagnostic methods could further enhance its sensitivity and specificity, offering a comprehensive approach to monitoring EH. Additionally, longitudinal studies tracking the progression of EH and its therapeutic responses will provide valuable insights into WAI’s potential as a diagnostic tool, bridging experimental findings with clinical practice.

## Conclusion

WAI demonstrates greater sensitivity than DPOAE in detecting frequency-specific acoustic changes associated with early-stage EH, emphasizing its potential as a non-invasive diagnostic tool for inner-ear disorders. By capturing alterations in middle-ear mechanics, such as increased stiffness and mass, WAI offers functional insights that complement traditional diagnostic methods. Future research should aim to validate WAI’s utility in both experimental and clinical contexts, refining its application for the early detection and management of inner-ear disorders like EH.

## Data Availability

The raw data supporting the conclusions of this article will be made available by the authors, without undue reservation.
