# Supplementary material for: The impact of endolymphatic hydrops on wideband acoustic immittance and otoacoustic emissions in guinea pigs
Source: Front Neurol. 2025 Jan 23;16:1444928. doi: 10.3389/fneur.2025.1444928 (PMC11800357; doi:10.3389/fneur.2025.1444928)
Supplement: Supplementary file 1 [file Table_1.pdf]

Supplementary Table 1: Justification for Vasopressin Dosage and Protocol

Study	Key Findings
Marshall et al. (2010)	Demonstrated that systemic vasopressin administration at similar doses reliably induced EH in guinea pig cochleae, confirmed through gadolinium-enhanced MRI imaging. Evidence of significant endolymphatic distension with minimal adverse effects supported this dosage as a reproducible model for EH studies.
Jiang et al. (2019)	Utilized desmopressin acetate to examine the AVP-aquaporin pathway in guinea pig EH models. Results confirmed effective endolymphatic fluid retention and associated biomechanical alterations in the cochlea. Validated this protocol for studying EH-induced fluid imbalances and their effects on cochlear mechanics.
Wang SQ et al. (2022)	Confirmed that the 10 μg/kg dosage successfully replicates EH-associated structural changes, making it suitable for exploring pathological and therapeutic conditions. Supported the protocol's reliability in generating measurable cochlear responses.
Wang C et al. (2022) - Our Prior Research	Employed the same DDAVP dosage to investigate molecular mechanisms in EH models. Revealed significant alterations in Epac1 and Epac2 signaling proteins, underscoring the pathway's role in cochlear fluid homeostasis. Validated the reproducibility of the dosage and demonstrated its utility in replicating EH for molecular and physiological studies.
Wang C et al. (2023) - Our Prior Research	Further confirmed the activation of cAMP-Epac1 signaling in DDAVP-induced EH models, emphasizing its relevance in cochlear fluid regulation. Highlighted the suitability of this dosage for studying the molecular mechanisms of EH.

The intraperitoneal administration of 10 μ g/kg desmopressin acetate (DDAVP) for 14 consecutive days was selected based on validated methodologies established in prior studies, including our team’s previous research. This dosage and timeline have consistently demonstrated efficacy in inducing endolymphatic hydrops (EH) in guinea pig models, providing a robust framework for investigating EH-related pathophysiology and acoustic changes. By replicating pathological features of EH, this protocol enables detailed exploration of acoustic transmission alterations, molecular pathways, and the evaluation of therapeutic strategies. The selected approach is both reproducible and ethically sound, ensuring reliable results for translational studies aimed at understanding and managing EH.

References

Marshall AF, Jewells VL, Kranz P, et al. Magnetic resonance imaging of guinea pig cochlea after vasopressin-induced or surgically induced endolymphatic hydrops. Otolaryngol Head Neck Surg. 2010;142(2):260-265.

Jiang L, He J, Chen X, et al. Arginine Vasopressin-Aquaporin-2 Pathway-Mediated Dehydration

Effects of Electroacupuncture in Guinea Pig Model of AVP-Induced Endolymphatic Hydrops. Chin J Integr Med. 2019;25(10):763-769.
Wang SQ, Li CL, Xu JQ, et al. The effect of endolymphatic hydrops and mannitol dehydration treatment on guinea pigs. Front Cell Neurosci. 2022; 16:836093.
Wang C, Li Y, Liu W, et al. The expression and significance of Epac1 and Epac2 in the inner ear of guinea pigs. Eur Arch Otorhinolaryngol. 2022;279(11):5207–5214.
Wang C, Li Y, Jiang W, et al. cAMP-Epac1 signaling is activated in DDAVP-induced endolymphatic hydrops of guinea pigs. Braz J Otorhinolaryngol. 2023;89(3):469-476.
